# Photophysical Properties of a Chiral Iridium-Based Photosensitizer as an Efficient Photodynamic Therapy Agent: A Theoretical Investigation

**DOI:** 10.3390/ijms26115062

**Published:** 2025-05-24

**Authors:** Maciej Spiegel

**Affiliations:** Department of Organic Chemistry and Pharmaceutical Technology, Wroclaw Medical University, Borowska 211 A, 50-556 Wroclaw, Poland; maciej.spiegel@umw.edu.pl

**Keywords:** photodynamic therapy, density functional theory, spin–orbit coupling, iridium complex, one-photon absorption, chirality, molecular modeling, reactive oxygen species, type I/II mechanisms

## Abstract

This study employs time-dependent density functional theory to explore the photophysical properties of a chiral iridium(III) complex designed as a photosensitizer for photodynamic therapy. Key properties analyzed include one-photon absorption energies, singlet–triplet energy gaps, spin–orbit coupling constants, and intersystem crossing rate constants. The potential for operation in a Type I PDT mechanism was assessed through ionization potential and electron affinity calculations. The results demonstrate that the complex is a promising PDT candidate, primarily operating in a Type II mechanism, while offering conditional viability for Type I photoreactivity under specific electronic and environmental conditions.

## 1. Introduction

The discovery of cisplatin’s anticancer properties marked a groundbreaking milestone in oncology, catalyzing extensive research into organometallic compounds aimed at reducing the side effects of conventional chemotherapy. Among emerging alternatives, photodynamic therapy (PDT) stands out as a minimally invasive technique that uses light to activate a photosensitizer, generating cytotoxic species such as singlet oxygen (^1^Δ_g_ O_2_). Beyond its applications in cancer treatment, PDT has shown promise in antibacterial, antiviral, and environmental contexts [1,2,3].

The photophysical mechanism of PDT involves the excitation of a photosensitizer within the therapeutic window (400–900 nm), followed by efficient intersystem crossing (ISC) from the first excited singlet state (S_1_) to a triplet state (T_n_). If the triplet state energy exceeds 0.98 eV, the energy transfer to ground-state triplet oxygen (^3^O_2_) produces cytotoxic ^1^Δ_g_ O_2_—a hallmark of the Type II mechanism. An ideal photosensitizer should meet several criteria: non-cytotoxicity in the dark, water solubility, redox stability, and strong absorption in the higher-frequency region of the therapeutic window for enhanced tissue penetration. Additionally, it should minimize fluorescence and exhibit significant spin–orbit coupling (SOC) to promote ISC—typically achieved through the incorporation of heavy metals into the molecular framework [4].

In photodynamic therapy, Type II mechanisms involve energy transfer from the excited triplet state of a photosensitizer to ground-state triplet oxygen, generating cytotoxic singlet oxygen (^1^O_2_). This pathway is oxygen-dependent and highly effective in well-oxygenated tissues. In contrast, Type I mechanisms proceed through electron or hydrogen atom transfer, forming reactive oxygen species (ROS) such as superoxide (O_2_^−•^) or hydroxyl radicals (^•^OH), and are more effective under hypoxic conditions. Each mechanism has distinct advantages and limitations. Type II offers high selectivity, predictable reactivity, and minimal side reactions but is ineffective in hypoxic tumors. In contrast, Type I can function under low-oxygen conditions and generate a broader spectrum of reactive oxygen species, although it may suffer from lower specificity, potential off-target effects, and dependence on cellular redox conditions. The ability of a single photosensitizer to access both mechanisms—depending on its electronic structure and environment—offers flexibility and therapeutic adaptability [5,6,7].

In recent years, complexes of ruthenium(II), iridium(III), and osmium(II) have been synthesized and characterized for their promising photophysical properties in PDT [8,9,10,11]. Notably, Wang et al. reported a pair of enantiopure mononuclear Ir(III) complexes to explore enantiomer-dependent anticancer activity [9]. Despite having identical structural and spectroscopic profiles, the enantiomers demonstrated different efficacies against specific cancer cell lines, indicating that chirality plays a critical role in therapeutic performance.

To elucidate the photophysical properties underlying PDT efficacy in such systems, this study employs density functional theory—a computational approach previously validated for analyzing second-row transition metal complexes in PDT research [12,13,14,15,16].

## 2. Results and Discussion

### 2.1. Structural Parameters

**Figure 1:**
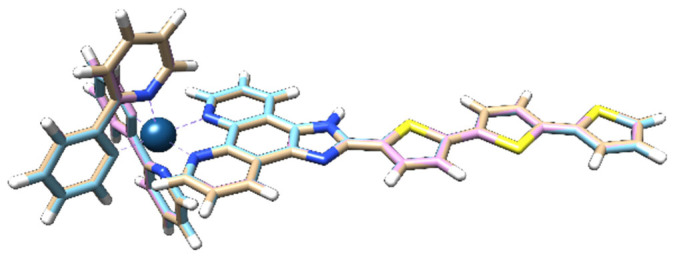
Superimposed structures of the ground state (S_0_, light brown), first excited singlet state (S_1_, cyan), and first excited triplet state (T_1_, pink) of the studied complex.

The optimized geometry of the iridium-based complex reveals a pseudo-octahedral coordination environment around the metal center. The electron-donating thiophene group is coplanar with the imidazo-phenanthroline moiety, while the other two thiophene rings deviate slightly from coplanarity. This configuration is preserved across the ground state (S_0_), first excited singlet (S_1_), and first excited triplet (T_1_), as shown in Figure 1.

The Ir–N bond lengths (Table 1) indicate that the bonds to the imidazo-phenanthroline nitrogens (D_1_, D_2_) are slightly longer than those of the phenylatopyridine ligands (D_3_, D_6_), which are, in turn, longer than the Ir-C bonds in the same ligands (D_4_, D_5_). These values are in close agreement with experimental X-ray data for similar iridium complexes, which report Ir–N distances of 2.159 Å (phenanthroline) and 2.040 Å (bipyridine) (bipyridine) [17], validating the computational model.

### 2.2. Excitation Energies and Absorption Spectra

**Figure 2:**
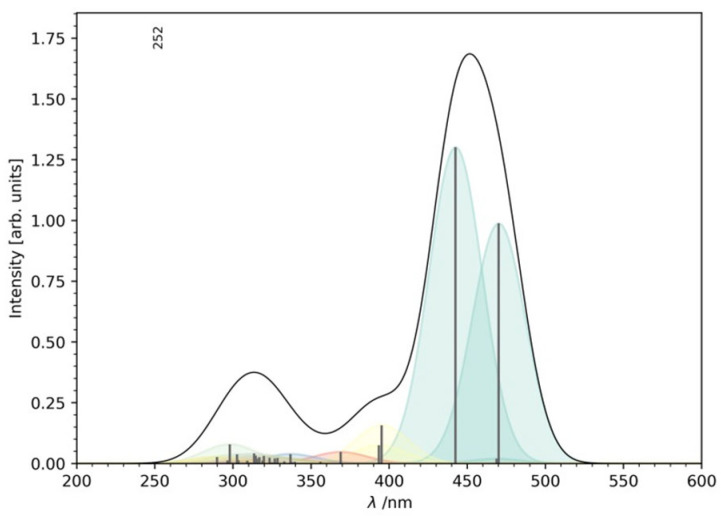
Simulated absorption spectrum of the studied complex.

**Figure 3:**
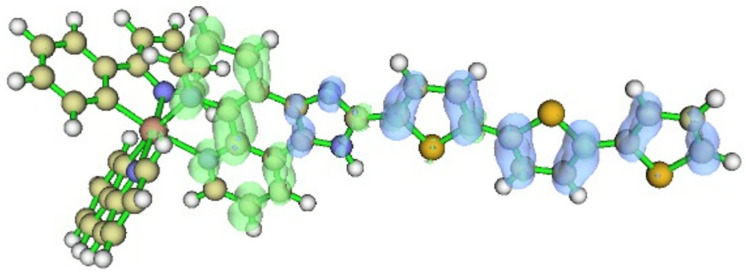
Hole (blue) and electron (green) charge transfer depiction for S_1_ excitation.

The computed vertical excitation energies (Table 2) and simulated absorption spectrum (Figure 2) indicate that the most intense absorption peak corresponds to the S_1_ excitation at 470 nm, aligning well with experimental observations. Charge transfer analysis (Figure 3) reveals an intra-ligand charge transfer from the thiophene moieties to the imidazo-phenanthroline core, a trend consistent with previous studies of transition metal complexes.

Three triplet states (T_1_–T_3_) lie below S_1_, each possessing energies exceeding the 0.98 eV threshold required for Type II PDT. The first triplet state (T_1_) at 658 nm also closely matches the experimental value of 647 nm, as determined by nanosecond time-resolved transient absorption spectroscopy [9]. This T_1_ transition is predominantly characterized by the highest occupied molecular orbital to the lowest unoccupied molecular orbital transition (H → L, 63.6%), with a secondary contribution from H → L + 1 (26.6%). The T_2_ state is primarily dominated by a H → L + 1 (52.5%) transition, with smaller contributions from H → L (25.5%) and H − 2 → L (10.4%). In the T_3_ state, the excitation consists mainly of nearly equal contributions from H − 1 → L and H − 1 → L + 1 transitions. Although T_3_ is nearly isoenergetic with S_1_, its optimized geometry exhibits slightly higher energy (by 0.1 eV).

### 2.3. Spin–Orbit Coupling and Intersystem Crossing

The spin–orbit coupling constants for the S_1_–T_1_, S_1_–T_1_ and S_1_–T_3_ intersystem crossing processes (Table 3), calculated at the optimized triplet geometries, are comparable to values reported for ruthenium-based complexes [18] and are sufficient to facilitate efficient ISC. The S_1_–T_3_ pathway, although slightly higher in energy than S_1_, is particularly favorable due to its large SOC value (317.76 cm⁻^1^), resulting in a high ISC rate constant (k_ISC_ = 3.71 × 10^9^ s⁻^1^). Interestingly, the S_1_–T_2_ intersystem crossing, despite its much lower SOC of 3.95 cm^−1^, still yields a substantial k_ISC_ of 1.77 × 10^9^ s⁻^1^, which is comparable in magnitude. This suggests that small energy gaps between states can compensate for low coupling strengths, enabling effective triplet population. The complex’s weak fluorescence, indicative of rapid ISC, supports these computational predictions and reinforces its suitability for PDT.

### 2.4. Type I Mechanism

As stated earlier, PDT can also proceed in Type I mechanisms, generating O_2_^−•^. Vertical electron affinities (VEA) and ionization potentials (VIP) were calculated as follows:

VEA:  −2.64 eV (Ps),  −4.18 eV (^3^Ps*),  −3.62 eV (^3^O_2_),

VIP:  5.28 eV (Ps),  3.66 eV (^3^Ps*).

The feasibility of the following autoionization reactions was evaluated:

^3^Ps* + Ps → Ps^+•^ + Ps^−•^, (1)

^3^Ps* + ^3^Ps* → Ps^+•^ + Ps^−•^. (2)

Reaction (2), which involves two triplet-state photosensitizers, is favorable due to its negative energy (−0.52 eV), computed as a sum of VIP (Ps) and VEA (^3^Ps*). In contrast, Reaction (1) is not favorable, yielding a positive energy (1.10 eV) from the sum of VIP and VEA of ^3^Ps*. The following subsequent reactions for O_2_^−•^ production were also considered:

Ps^−•^ + ^3^O_2_ → Ps + O_2_^−•^, (3)

^3^Ps* + ^3^O_2_ → Ps^+•^ + O_2_^−•^. (4)

Reaction (3) is thermodynamically feasible, with an energy change of −0.98 eV (VEA (^3^O_2_) + [−VIP (Ps)]). Reaction (4), on the other hand, results in a slightly positive energy change (0.04 eV), suggesting it is less favorable, though potential numerical uncertainties may still allow it to occur. Thus, the production of O_2_^−•^ is most likely from the Reaction (3) and overall outcomes support a plausible Type I mechanism under certain conditions.

In summary, the complex is expected to operate primarily through a Type II mechanism under normoxic conditions due to its efficient singlet oxygen generation. However, under hypoxic conditions—where O_2_ availability is limited—or in electron-rich environments [19], such as cells with high metabolic activity or the presence of reducing biomolecules like thiols, phenols, or other electron donors, Type I processes become favored, including the formation of superoxide through electron transfer from the excited triplet state [20].

## 3. Materials and Methods

Geometry optimizations for both isomeric forms of the complex were performed using the Orca 6.0 software package [21]. Calculations employed the B3LYP functional formulated in the Gaussian way [22,23], along with the x2c-SVPall basis set, within the exact two-component (X2C) relativistic Hamiltonian framework [24] to account for relativistic effects. Solvent interactions (acetonitrile) were modeled using the SMD implicit solvation approach [25], and dispersion interactions were corrected using Grimme’s D4 empirical method [26].

All optimized geometries were confirmed as true minima by vibrational frequency analysis, which showed no imaginary frequencies. Spin–orbit coupling (SOC) elements were calculated at the optimized geometry of the T_1_ state. These data—including geometries, Hessians, and SOC matrices—were subsequently used in excited-state dynamics simulations to determine intersystem crossing and fluorescence rate constants. Charge transfer behavior was analyzed through hole–electron decomposition using Multiwfn 3.8 [27,28] while molecular visualizations were generated with UCSF Chimera 1.19 [29].

## 4. Conclusions

This DFT-based quantum chemical investigation of a recently synthesized chiral iridium(III) imidazo-phenanthroline complex provides compelling evidence of its potential as an effective photosensitizer for photodynamic therapy. The key findings of this study are summarized as follows:

- The optimized geometrical parameters, particularly the Ir-N bond lengths, are in good agreement with experimental X-ray crystallographic data for analogous iridium complexes, validating the computational approach.
- The calculated vertical excitation energies accurately reproduce the experimental absorption spectrum. Notably, the S_1_ absorption peak at ~470 nm is attributed to an intra-ligand charge transfer from the thiophene substituents to the imidazo-phenanthroline core, consistent with observed optical behavior.
- Spin–orbit coupling between singlet and triplet states, particularly in the S_1_–T_2_ and S_1_–T_3_ pathways, is sufficient to enable rapid intersystem crossing. This supports an efficient Type II PDT mechanism via singlet oxygen generation.
- Thermodynamic analysis indicates that electron transfer leading to superoxide formation is feasible under certain conditions—specifically when the photosensitizer is in the singlet excited state—suggesting that both Type I and Type II PDT mechanisms are accessible.
- These results position the chiral iridium complex as a versatile and promising candidate for PDT applications. Future work will focus on experimental validation, phototoxicity assessments, and structural optimization to enhance therapeutic performance and selectivity.

## Figures and Tables

**Table 1:** Ir–N bond lengths (Å) for ground state (S_0_), first excited singlet (S_1_), and first excited triplet (T_1_).

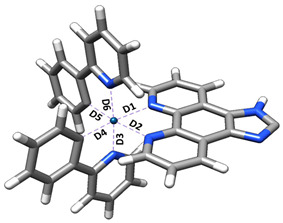						
State	D_1_	D_2_	D_3_	D_4_	D_5_	D_6_
S_0_	2.187	2.187	2.070	2.016	2.016	2.070
S_1_	2.156	2.152	2.054	2.011	2.011	2.054
T_1_	2.157	2.156	2.055	2.010	2.010	2.055

**Table 2:** Vertical excitation energies (ΔE, eV), wavelengths (λ, nm), oscillator strengths (f), and main transitions (%) in dichloromethane.

State	E	λ	*f*	Transition (%)
T_1_	1.88	658.0	0.997	H → L (63.6%) H → L + 1 (26.6%)
T_2_	2.50	495.6	0.997	H → L (25.5%) H → L + 1 (52.5%) H − 2 → L (10.4%)
T_3_	2.61	474.3	0.997	H − 1 → L (44.8%) H − 1 → L + 1 (41.6%)
S_1_	2.64	470.0	0.997	H → L (86.6%) H → L + 1 (9.1%)

**Table 3:** Computed energy gaps (Δ*E_(S₁-Tⱼ)_*, in eV), spin–orbit couplings (SOCs, in cm^−1^), and intersystem crossing rate constants (*k*_ISC_, in s^−1^).

T	Δ*E_(S₁-Tⱼ)_*	SOC	k_ISC_
T_1_	0.74	0.28	5.73 × 10^2^
T_2_	0.03	3.95	1.77 × 10^9^
T_3_	−0.07	217.76	3.71 × 10^9^

## Data Availability

The data presented in this study are available on request from the corresponding author.
